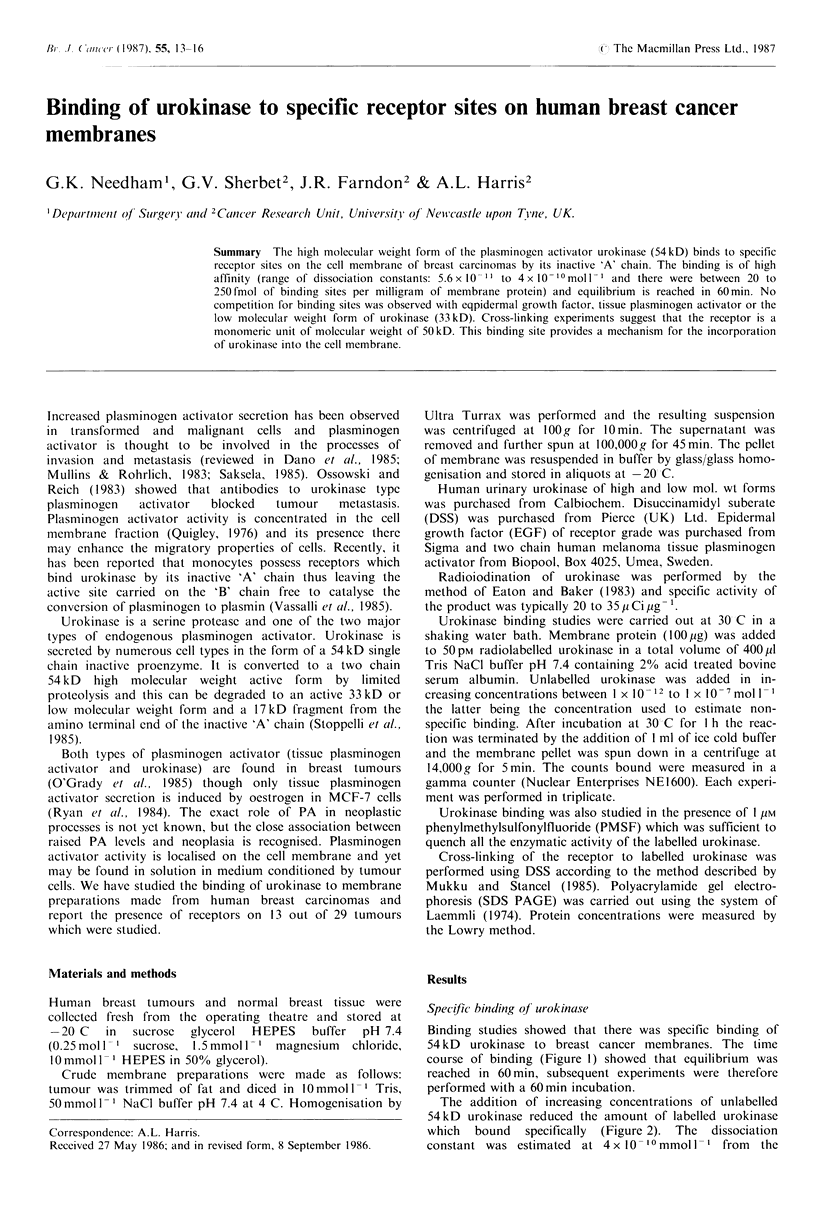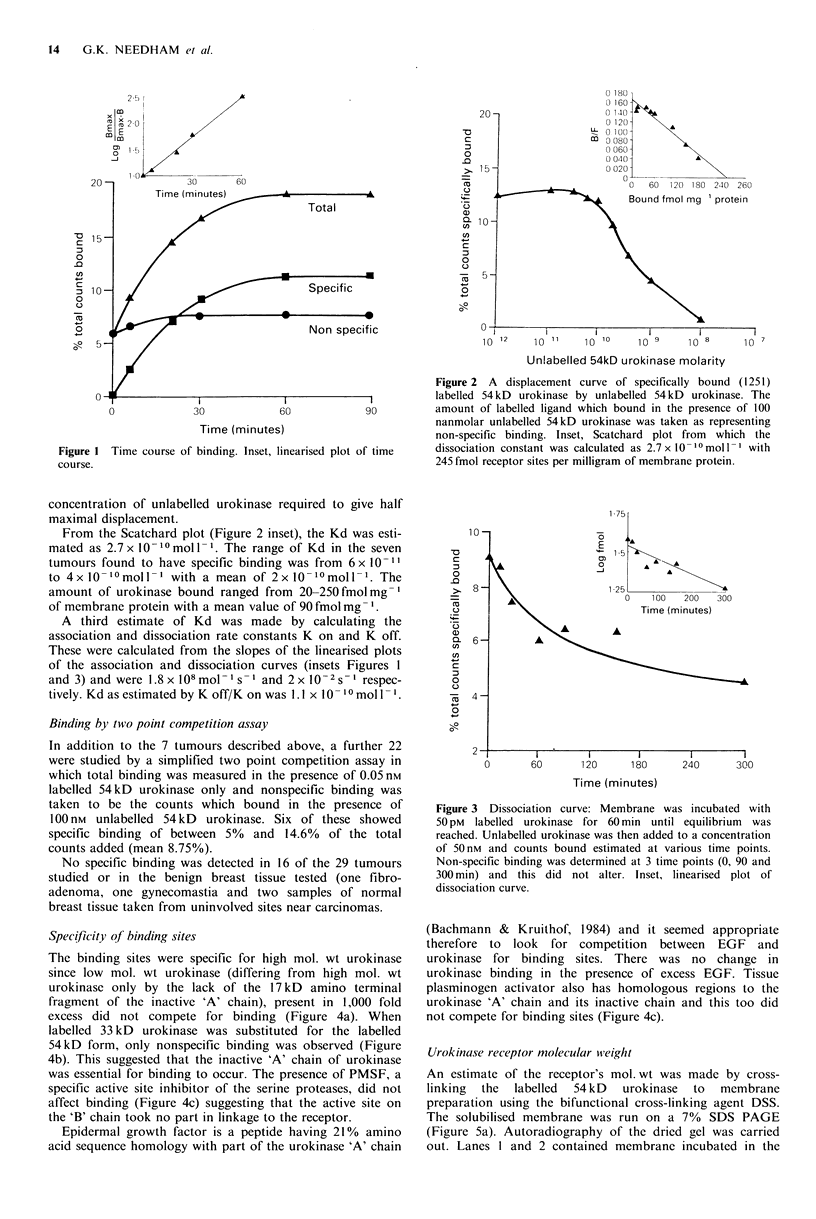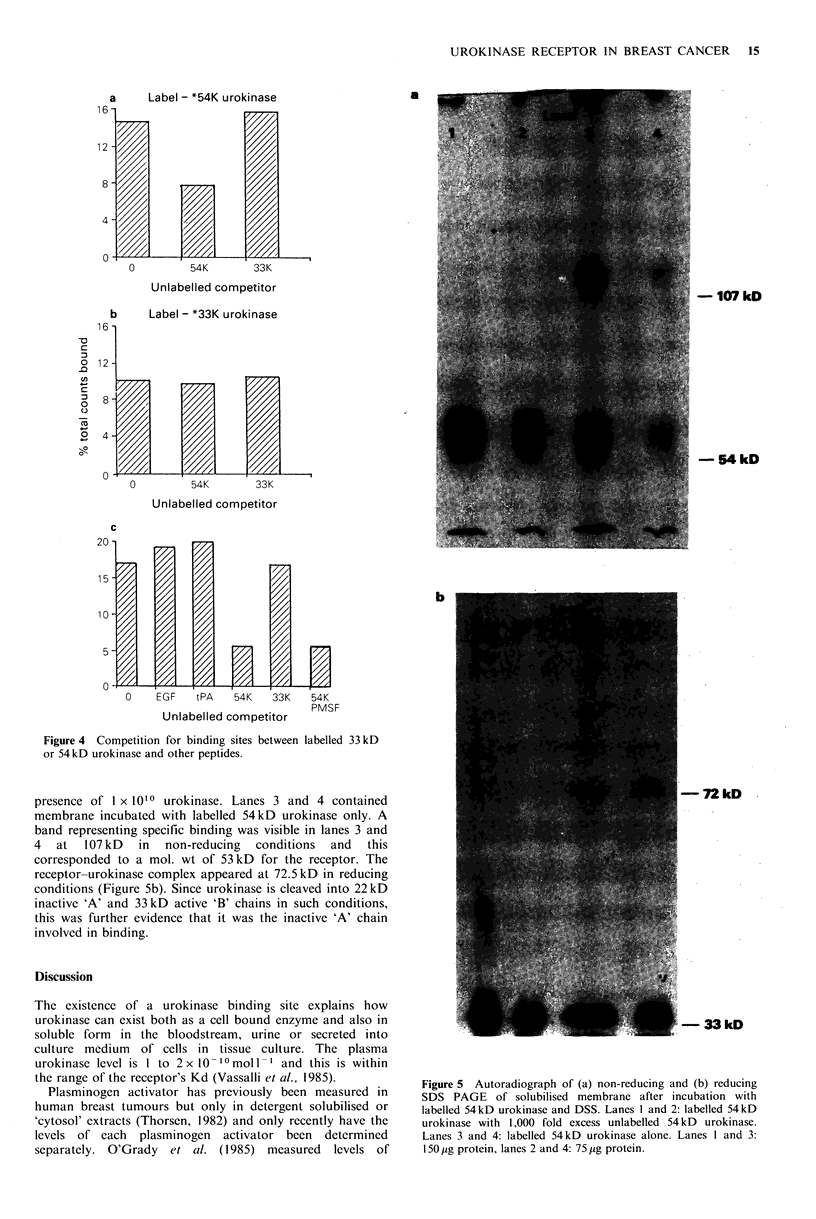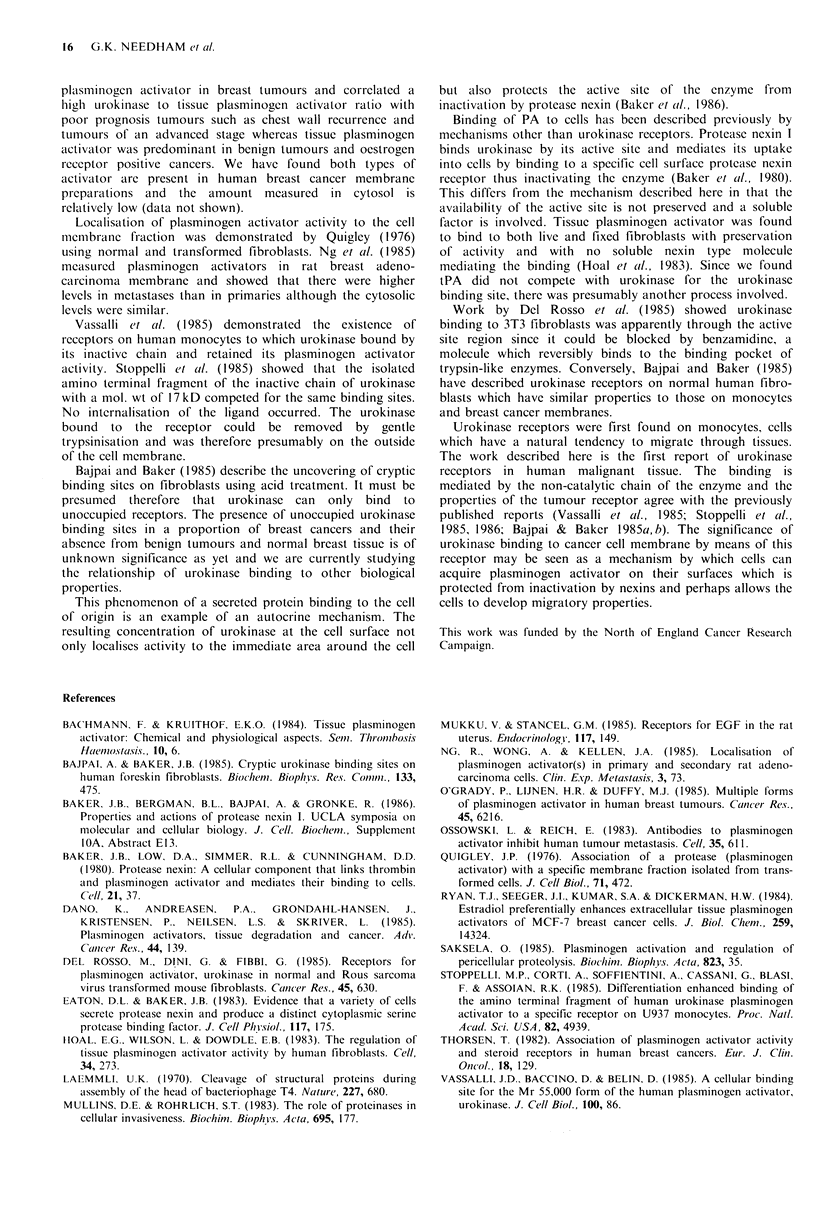# Binding of urokinase to specific receptor sites on human breast cancer membranes.

**DOI:** 10.1038/bjc.1987.3

**Published:** 1987-01

**Authors:** G. K. Needham, G. V. Sherbet, J. R. Farndon, A. L. Harris

## Abstract

**Images:**


Br. J. Cancer (1987), 55, 13-16 © The Macmillan Press Ltd., 1987

### Binding of urokinase to specific receptor sites on human breast cancer membranes

G.K. Needham[1], G.V. Sherbet[2], J.R. Farndon[2] & A.L. Harris[2]

[1]Department of Surgery and [2]Cancer Research Unit, University of Newcastle upon Tyne, UK.

**Summary** The high molecular weight form of the plasminogen activator urokinase (54 kD) binds to specific receptor sites on the cell membrane of breast carcinomas by its inactive 'A' chain. The binding is of high affinity (range of dissociation constants: $5.6\times10^{-11}$ to $4\times10^{-10}$ mol l$^{-1}$ and there were between 20 to 250 fmol of binding sites per milligram of membrane protein) and equilibrium is reached in 60 min. No competition for binding sites was observed with eqpidermal growth factor, tissue plasminogen activator or the low molecular weight form of urokinase (33 kD). Cross-linking experiments suggest that the receptor is a monomeric unit of molecular weight of 50 kD. This binding site provides a mechanism for the incorporation of urokinase into the cell membrane.

Increased plasminogen activator secretion has been observed in transformed and malignant cells and plasminogen activator is thought to be involved in the processes of invasion and metastasis (reviewed in Dano *et al.*, 1985; Mullins & Rohrlich, 1983; Saksela, 1985). Ossowski and Reich (1983) showed that antibodies to urokinase type plasminogen activator blocked tumour metastasis. Plasminogen activator activity is concentrated in the cell membrane fraction (Quigley, 1976) and its presence there may enhance the migratory properties of cells. Recently, it has been reported that monocytes possess receptors which bind urokinase by its inactive 'A' chain thus leaving the active site carried on the 'B' chain free to catalyse the conversion of plasminogen to plasmin (Vassalli *et al.*, 1985).

Urokinase is a serine protease and one of the two major types of endogenous plasminogen activator. Urokinase is secreted by numerous cell types in the form of a 54 kD single chain inactive proenzyme. It is converted to a two chain 54 kD high molecular weight active form by limited proteolysis and this can be degraded to an active 33 kD or low molecular weight form and a 17 kD fragment from the amino terminal end of the inactive 'A' chain (Stoppelli *et al.*, 1985).

Both types of plasminogen activator (tissue plasminogen activator and urokinase) are found in breast tumours (O'Grady *et al.*, 1985) though only tissue plasminogen activator secretion is induced by oestrogen in MCF-7 cells (Ryan *et al.*, 1984). The exact role of PA in neoplastic processes is not yet known, but the close association between raised PA levels and neoplasia is recognised. Plasminogen activator activity is localised on the cell membrane and yet may be found in solution in medium conditioned by tumour cells. We have studied the binding of urokinase to membrane preparations made from human breast carcinomas and report the presence of receptors on 13 out of 29 tumours which were studied.

### Materials and methods

Human breast tumours and normal breast tissue were collected fresh from the operating theatre and stored at −20°C in sucrose glycerol HEPES buffer pH 7.4 (0.25 mol l$^{-1}$ sucrose, 1.5 mmol l$^{-1}$ magnesium chloride, 10 mmol l$^{-1}$ HEPES in 50% glycerol).

Crude membrane preparations were made as follows: tumour was trimmed of fat and diced in 10 mmol l$^{-1}$ Tris, 50 mmol l$^{-1}$ NaCl buffer pH 7.4 at 4°C. Homogenisation by Ultra Turrax was performed and the resulting suspension was centrifuged at 100 *g* for 10 min. The supernatant was removed and further spun at 100,000 *g* for 45 min. The pellet of membrane was resuspended in buffer by glass/glass homogenisation and stored in aliquots at −20°C.

Human urinary urokinase of high and low mol. wt forms was purchased from Calbiochem. Disuccinamidyl suberate (DSS) was purchased from Pierce (UK) Ltd. Epidermal growth factor (EGF) of receptor grade was purchased from Sigma and two chain human melanoma tissue plasminogen activator from Biopool, Box 4025, Umea, Sweden.

Radioiodination of urokinase was performed by the method of Eaton and Baker (1983) and specific activity of the product was typically 20 to 35 $\mu$Ci $\mu$g$^{-1}$.

Urokinase binding studies were carried out at 30°C in a shaking water bath. Membrane protein (100 $\mu$g) was added to 50 pM radiolabelled urokinase in a total volume of 400 $\mu$l Tris NaCl buffer pH 7.4 containing 2% acid treated bovine serum albumin. Unlabelled urokinase was added in increasing concentrations between $1\times10^{-12}$ to $1\times10^{-7}$ mol l$^{-1}$ the latter being the concentration used to estimate non-specific binding. After incubation at 30°C for 1 h the reaction was terminated by the addition of 1 ml of ice cold buffer and the membrane pellet was spun down in a centrifuge at 14,000 *g* for 5 min. The counts bound were measured in a gamma counter (Nuclear Enterprises NE1600). Each experiment was performed in triplicate.

Urokinase binding was also studied in the presence of 1 $\mu$M phenylmethylsulfonylfluoride (PMSF) which was sufficient to quench all the enzymatic activity of the labelled urokinase.

Cross-linking of the receptor to labelled urokinase was performed using DSS according to the method described by Mukku and Stancel (1985). Polyacrylamide gel electrophoresis (SDS PAGE) was carried out using the system of Laemmli (1974). Protein concentrations were measured by the Lowry method.

### Results

### *Specific binding of urokinase*

Binding studies showed that there was specific binding of 54 kD urokinase to breast cancer membranes. The time course of binding (Figure 1) showed that equilibrium was reached in 60 min, subsequent experiments were therefore performed with a 60 min incubation.

The addition of increasing concentrations of unlabelled 54 kD urokinase reduced the amount of labelled urokinase which bound specifically (Figure 2). The dissociation constant was estimated at $4\times10^{-10}$ mmol l$^{-1}$ from the

Correspondence: A.L. Harris.
Received 27 May 1986; and in revised form, 8 September 1986.

**Figure 1** Time course of binding. Inset, linearised plot of time course.

**Figure 2** A displacement curve of specifically bound (1251) labelled 54 kD urokinase by unlabelled 54 kD urokinase. The amount of labelled ligand which bound in the presence of 100 nanmolar unlabelled 54 kD urokinase was taken as representing non-specific binding. Inset, Scatchard plot from which the dissociation constant was calculated as $2.7 \times 10^{-10}$ mol l$^{-1}$ with 245 fmol receptor sites per milligram of membrane protein.

concentration of unlabelled urokinase required to give half maximal displacement.

From the Scatchard plot (Figure 2 inset), the Kd was estimated as $2.7 \times 10^{-10}$ mol l$^{-1}$. The range of Kd in the seven tumours found to have specific binding was from $6 \times 10^{-11}$ to $4 \times 10^{-10}$ mol l$^{-1}$ with a mean of $2 \times 10^{-10}$ mol l$^{-1}$. The amount of urokinase bound ranged from 20–250 fmol mg$^{-1}$ of membrane protein with a mean value of 90 fmol mg$^{-1}$.

A third estimate of Kd was made by calculating the association and dissociation rate constants K on and K off. These were calculated from the slopes of the linearised plots of the association and dissociation curves (insets Figures 1 and 3) and were $1.8 \times 10^{8}$ mol$^{-1}$ s$^{-1}$ and $2 \times 10^{-2}$ s$^{-1}$ respectively. Kd as estimated by K off/K on was $1.1 \times 10^{-10}$ mol l$^{-1}$.

*Binding by two point competition assay*

In addition to the 7 tumours described above, a further 22 were studied by a simplified two point competition assay in which total binding was measured in the presence of 0.05 nM labelled 54 kD urokinase only and nonspecific binding was taken to be the counts which bound in the presence of 100 nM unlabelled 54 kD urokinase. Six of these showed specific binding of between 5% and 14.6% of the total counts added (mean 8.75%).

No specific binding was detected in 16 of the 29 tumours studied or in the benign breast tissue tested (one fibroadenoma, one gynecomastia and two samples of normal breast tissue taken from uninvolved sites near carcinomas.

*Specificity of binding sites*

The binding sites were specific for high mol. wt urokinase since low mol. wt urokinase (differing from high mol. wt urokinase only by the lack of the 17 kD amino terminal fragment of the inactive 'A' chain), present in 1,000 fold excess did not compete for binding (Figure 4a). When labelled 33 kD urokinase was substituted for the labelled 54 kD form, only nonspecific binding was observed (Figure 4b). This suggested that the inactive 'A' chain of urokinase was essential for binding to occur. The presence of PMSF, a specific active site inhibitor of the serine proteases, did not affect binding (Figure 4c) suggesting that the active site on the 'B' chain took no part in linkage to the receptor.

Epidermal growth factor is a peptide having 21% amino acid sequence homology with part of the urokinase 'A' chain

**Figure 3** Dissociation curve: Membrane was incubated with 50 pM labelled urokinase for 60 min until equilibrium was reached. Unlabelled urokinase was then added to a concentration of 50 nM and counts bound estimated at various time points. Non-specific binding was determined at 3 time points (0, 90 and 300 min) and this did not alter. Inset, linearised plot of dissociation curve.

(Bachmann & Kruithof, 1984) and it seemed appropriate therefore to look for competition between EGF and urokinase for binding sites. There was no change in urokinase binding in the presence of excess EGF. Tissue plasminogen activator also has homologous regions to the urokinase 'A' chain and its inactive chain and this too did not compete for binding sites (Figure 4c).

*Urokinase receptor molecular weight*

An estimate of the receptor's mol. wt was made by cross-linking the labelled 54 kD urokinase to membrane preparation using the bifunctional cross-linking agent DSS. The solubilised membrane was run on a 7% SDS PAGE (Figure 5a). Autoradiography of the dried gel was carried out. Lanes 1 and 2 contained membrane incubated in the

**Figure 4** Competition for binding sites between labelled 33 kD or 54 kD urokinase and other peptides.

presence of $1 \times 10^{10}$ urokinase. Lanes 3 and 4 contained membrane incubated with labelled 54 kD urokinase only. A band representing specific binding was visible in lanes 3 and 4 at 107 kD in non-reducing conditions and this corresponded to a mol. wt of 53 kD for the receptor. The receptor–urokinase complex appeared at 72.5 kD in reducing conditions (Figure 5b). Since urokinase is cleaved into 22 kD inactive 'A' and 33 kD active 'B' chains in such conditions, this was further evidence that it was the inactive 'A' chain involved in binding.

### Discussion

The existence of a urokinase binding site explains how urokinase can exist both as a cell bound enzyme and also in soluble form in the bloodstream, urine or secreted into culture medium of cells in tissue culture. The plasma urokinase level is 1 to $2 \times 10^{-10}$ mol l$^{-1}$ and this is within the range of the receptor's Kd (Vassalli *et al.*, 1985).

Plasminogen activator has previously been measured in human breast tumours but only in detergent solubilised or 'cytosol' extracts (Thorsen, 1982) and only recently have the levels of each plasminogen activator been determined separately. O'Grady *et al.* (1985) measured levels of

**Figure 5** Autoradiograph of (a) non-reducing and (b) reducing SDS PAGE of solubilised membrane after incubation with labelled 54 kD urokinase and DSS. Lanes 1 and 2: labelled 54 kD urokinase with 1,000 fold excess unlabelled 54 kD urokinase. Lanes 3 and 4: labelled 54 kD urokinase alone. Lanes 1 and 3: 150 $\mu$g protein, lanes 2 and 4: 75 $\mu$g protein.

plasminogen activator in breast tumours and correlated a high urokinase to tissue plasminogen activator ratio with poor prognosis tumours such as chest wall recurrence and tumours of an advanced stage whereas tissue plasminogen activator was predominant in benign tumours and oestrogen receptor positive cancers. We have found both types of activator are present in human breast cancer membrane preparations and the amount measured in cytosol is relatively low (data not shown).

Localisation of plasminogen activator activity to the cell membrane fraction was demonstrated by Quigley (1976) using normal and transformed fibroblasts. Ng *et al.* (1985) measured plasminogen activators in rat breast adenocarcinoma membrane and showed that there were higher levels in metastases than in primaries although the cytosolic levels were similar.

Vassalli *et al.* (1985) demonstrated the existence of receptors on human monocytes to which urokinase bound by its inactive chain and retained its plasminogen activator activity. Stoppelli *et al.* (1985) showed that the isolated amino terminal fragment of the inactive chain of urokinase with a mol. wt of 17 kD competed for the same binding sites. No internalisation of the ligand occurred. The urokinase bound to the receptor could be removed by gentle trypsinisation and was therefore presumably on the outside of the cell membrane.

Bajpai and Baker (1985) describe the uncovering of cryptic binding sites on fibroblasts using acid treatment. It must be presumed therefore that urokinase can only bind to unoccupied receptors. The presence of unoccupied urokinase binding sites in a proportion of breast cancers and their absence from benign tumours and normal breast tissue is of unknown significance as yet and we are currently studying the relationship of urokinase binding to other biological properties.

This phenomenon of a secreted protein binding to the cell of origin is an example of an autocrine mechanism. The resulting concentration of urokinase at the cell surface not only localises activity to the immediate area around the cell but also protects the active site of the enzyme from inactivation by protease nexin (Baker *et al.*, 1986).

Binding of PA to cells has been described previously by mechanisms other than urokinase receptors. Protease nexin I binds urokinase by its active site and mediates its uptake into cells by binding to a specific cell surface protease nexin receptor thus inactivating the enzyme (Baker *et al.*, 1980). This differs from the mechanism described here in that the availability of the active site is not preserved and a soluble factor is involved. Tissue plasminogen activator was found to bind to both live and fixed fibroblasts with preservation of activity and with no soluble nexin type molecule mediating the binding (Hoal *et al.*, 1983). Since we found tPA did not compete with urokinase for the urokinase binding site, there was presumably another process involved.

Work by Del Rosso *et al.* (1985) showed urokinase binding to 3T3 fibroblasts was apparently through the active site region since it could be blocked by benzamidine, a molecule which reversibly binds to the binding pocket of trypsin-like enzymes. Conversely, Bajpai and Baker (1985) have described urokinase receptors on normal human fibroblasts which have similar properties to those on monocytes and breast cancer membranes.

Urokinase receptors were first found on monocytes, cells which have a natural tendency to migrate through tissues. The work described here is the first report of urokinase receptors in human malignant tissue. The binding is mediated by the non-catalytic chain of the enzyme and the properties of the tumour receptor agree with the previously published reports (Vassalli *et al.*, 1985; Stoppelli *et al.*, 1985, 1986; Bajpai & Baker 1985*a,b*). The significance of urokinase binding to cancer cell membrane by means of this receptor may be seen as a mechanism by which cells can acquire plasminogen activator on their surfaces which is protected from inactivation by nexins and perhaps allows the cells to develop migratory properties.

This work was funded by the North of England Cancer Research Campaign.

### References

BACHMANN, F. & KRUITHOF, E.K.O. (1984). Tissue plasminogen activator: Chemical and physiological aspects. *Sem. Thrombosis Haemostasis.*, **10**, 6.

BAJPAI, A. & BAKER, J.B. (1985). Cryptic urokinase binding sites on human foreskin fibroblasts. *Biochem. Biophys. Res. Comm.*, **133**, 475.

BAKER, J.B., BERGMAN, B.L., BAJPAI, A. & GRONKE, R. (1986). Properties and actions of protease nexin I. UCLA symposia on molecular and cellular biology. *J. Cell. Biochem.*, Supplement 10A, Abstract E13.

BAKER, J.B., LOW, D.A., SIMMER, R.L. & CUNNINGHAM, D.D. (1980). Protease nexin: A cellular component that links thrombin and plasminogen activator and mediates their binding to cells. *Cell*, **21**, 37.

DANO, K., ANDREASEN, P.A., GRONDAHL-HANSEN, J., KRISTENSEN, P., NEILSEN, L.S. & SKRIVER, L. (1985). Plasminogen activators, tissue degradation and cancer. *Adv. Cancer Res.*, **44**, 139.

DEL ROSSO, M., DINI, G. & FIBBI, G. (1985). Receptors for plasminogen activator, urokinase in normal and Rous sarcoma virus transformed mouse fibroblasts. *Cancer Res.*, **45**, 630.

EATON, D.L. & BAKER, J.B. (1983). Evidence that a variety of cells secrete protease nexin and produce a distinct cytoplasmic serine protease binding factor. *J. Cell Physiol.*, **117**, 175.

HOAL, E.G., WILSON, L. & DOWDLE, E.B. (1983). The regulation of tissue plasminogen activator activity by human fibroblasts. *Cell*, **34**, 273.

LAEMMLI, U.K. (1970). Cleavage of structural proteins during assembly of the head of bacteriophage T4. *Nature*, **227**, 680.

MULLINS, D.E. & ROHRLICH, S.T. (1983). The role of proteinases in cellular invasiveness. *Biochim. Biophys. Acta*, **695**, 177.

MUKKU, V. & STANCEL, G.M. (1985). Receptors for EGF in the rat uterus. *Endocrinology*, **117**, 149.

NG, R., WONG, A. & KELLEN, J.A. (1985). Localisation of plasminogen activator(s) in primary and secondary rat adenocarcinoma cells. *Clin. Exp. Metastasis*, **3**, 73.

O'GRADY, P., LIJNEN, H.R. & DUFFY, M.J. (1985). Multiple forms of plasminogen activator in human breast tumours. *Cancer Res.*, **45**, 6216.

OSSOWSKI, L. & REICH, E. (1983). Antibodies to plasminogen activator inhibit human tumour metastasis. *Cell*, **35**, 611.

QUIGLEY, J.P. (1976). Association of a protease (plasminogen activator) with a specific membrane fraction isolated from transformed cells. *J. Cell Biol.*, **71**, 472.

RYAN, T.J., SEEGER, J.I., KUMAR, S.A. & DICKERMAN, H.W. (1984). Estradiol preferentially enhances extracellular tissue plasminogen activators of MCF-7 breast cancer cells. *J. Biol. Chem.*, **259**, 14324.

SAKSELA, O. (1985). Plasminogen activation and regulation of pericellular proteolysis. *Biochim. Biophys. Acta*, **823**, 35.

STOPPELLI, M.P., CORTI, A., SOFFIENTINI, A., CASSANI, G., BLASI, F. & ASSOIAN, R.K. (1985). Differentiation enhanced binding of the amino terminal fragment of human urokinase plasminogen activator to a specific receptor on U937 monocytes. *Proc. Natl. Acad. Sci. USA*, **82**, 4939.

THORSEN, T. (1982). Association of plasminogen activator activity and steroid receptors in human breast cancers. *Eur. J. Clin. Oncol.*, **18**, 129.

VASSALLI, J.D., BACCINO, D. & BELIN, D. (1985). A cellular binding site for the Mr 55,000 form of the human plasminogen activator, urokinase. *J. Cell Biol.*, **100**, 86.